# Effects of Dietary Flavonoids on Mood and Mental Health: A Systematic Review

**DOI:** 10.1093/nutrit/nuaf188

**Published:** 2025-11-14

**Authors:** Rebecca L Colombage, Katie L Barfoot, Daniel J Lamport

**Affiliations:** School of Psychology and Clinical Language Sciences, University of Reading, Reading, United Kingdom; School of Psychology and Clinical Language Sciences, University of Reading, Reading, United Kingdom; School of Psychology and Clinical Language Sciences, University of Reading, Reading, United Kingdom

**Keywords:** diet, flavonoids, mood, mental health

## Abstract

**Context:**

Collective evidence has highlighted the strong interplay of the diet with mood and mental health. The diet is a topic of interest from a public health perspective as it may provide an adoptable lifestyle approach for improving mental health in the population. Among dietary constituents, flavonoids have been identified as particularly relevant for mental health.

**Objective:**

The aim of this review was to evaluate the effects of dietary flavonoids on mental health in healthy populations across the lifespan.

**Data sources:**

Experimental human trials with study participants of any age, sex, or ethnicity were eligible for inclusion in this review if they included supplementation with at least 1 flavonoid-rich food (<15 mg/100 g/mL flavonoid constituents) either as lyophilized powder or whole food and measured at least 1 mood outcome. The PubMed, Web of Science, and Scopus databases were searched with no restriction on publication start date to October 2024. The Evidence Analysis Manual Quality Criteria Checklist (QCC) from the Academy of Nutrition and Dietetics Evidence Analysis Library^®^ was used to assess methodology and risk of bias.

**Data extraction:**

A total of 38 experimental studies met eligibility criteria, 13 exploring acute effects of flavonoids and 25 utilising a chronic design.

**Data analysis:**

The included studies explored a range of flavonoid-containing foods, with a majority (*n* = 9) utilizing cocoa as an intervention vehicle. Five of the 13 acute studies reported benefits (3 wild blueberry, 1 purple grape juice, 1 orange juice); and 12 of 25 chronic studies showed findings in favor of flavonoid supplementation (2 cocoa, 2 blueberry, 2 cherry, 1 peppermint, 1 orange juice, 1 walnut, 1 green tea, and 2 mixed foods), suggesting chronic supplementation may benefit mood and mental health.

**Conclusions:**

Further studies are required to understand the effects of dietary flavonoids utilizing consistent methodology and dosing, as well as to explore mechanistic links with mental health across the lifespan.

**Systematic Review Registration:**

PROSPERO registration No. [CRD42021293040].

## INTRODUCTION

Mental health problems have emerged as a pressing and increasingly prevalent concern worldwide, with depression and anxiety standing out as significant contributors to global health-related pressures. Since the COVID-19 pandemic, major depressive and anxiety disorders have been estimated to have had an additional 53.2 million cases and 76.2 million cases, respectively.^1^ Psychotropic medications including antidepressants and anxiolytic medications are typically used in treatments for mood disorders and are often used in conjunction with talking therapies.^2^ However, these treatment approaches have limitations, such as delayed onset and side effects.^3^^,^^4^ Importantly, not all patients diagnosed with mood disorders have access to treatments, and 76.3% to 85.4% of those in less developed countries do not receive adequate treatment.^5^ Moreover, even in patients for whom treatment availability is good, Jorm et al. (2017)^6^ highlight challenges associated with the implementation of these treatments, such as access to care and individual variability and adherence. Collectively, this emphasises a need to further explore adjunctive treatments that are more accessible and cost effective for all.

Evidence from observation and intervention studies shows that lifestyle factors such as diet, in particular diets abundant in fruits and vegetables, may play a mediating role in the treatment of mood disorders.^7^ Recently, O’Neil et al. (2024),^8^ when considering both clinical and cost-effective outcomes, found that lifestyle interventions that target nutrition and physical activity are effective, which suggests suitable treatment alternatives or additions to traditional therapies for mood disorders. A previous review^9^ summarized results from 61 studies and highlighted the finding that total fruit and vegetable intake may promote higher levels of optimism and self-efficacy as well as reducing psychological distress. It was further suggested that consumption of at least 5 portions per day is likely to lead to better outcomes, with certain subgroups of fruits and vegetables having the most impact on mood, such as berries, citrus fruits, and leafy green vegetables. This finding of key subgroups of fruits and vegetables is not standalone. For example, Sun et al. (2021)^10^ conducted a cross-sectional study of 16 925 adults with 24-hour dietary recalls, and found that higher intakes of tomatoes, dark green vegetables, and berries were inversely related to depressive symptoms.

The findings of the cross-sectional work have also been validated in experimental trials. In a randomized controlled trial, 171 young adults (age 18-25 years) with low fruit and vegetable consumption were given 2 additional daily servings of fruit and vegetables in addition to their normal diet for 14 days. Results showed improved psychological wellbeing with increased motivation, vitality, and flourishing (defined as self-reported curiosity, creativity, and motivation) in the experimental subjects compared to controls.^11^ Additionally, Kontogianni et al. (2020)^12^ found that in 99 mildly hypertensive patients supplementation with 6 portions of fruit and vegetables per day, including 1 portion of berries and 50 g dark chocolate for 8 weeks led to a decrease in depressive symptoms. These results provide evidence not only for the benefits of fruits and vegetables but also other flavonoid-rich foods (dark chocolate and berries), as well as highlighting the finding that certain populations, such as hypertensive adults, may be more sensitive to flavonoid interventions.

Due to potential antidepressant and anxiolytic properties, flavonoids, a class of compounds within the polyphenol family, have attracted considerable attention in the treatment of mood disorders. This relationship has been explored recently in several epidemiological studies: Mestrom et al. (2024)^13^ found that study participants with major depressive disorder (MDD) had significantly lower intakes of anthocyanins than participants without MDD. This finding has also been supported with cross-sectional data from the NHANES cohort, highlighting a negative association between anthocyanidin intake and depression.^14^ Together, these studies highlight the finding that certain subtypes of flavonoids may provide the greatest benefit when consumed habitually over a lifetime.

Mechanistically, flavonoids have been shown to cross the blood–brain barrier and act as monoamine oxidase (MAO) inhibitors,^15^^,^^16^ which break down serotonin, dopamine, and noradrenaline in the brain. Hence inhibition of this action via flavonoid supplementation is likely to benefit mood and mental health.^17^ Neuroplasticity is another mechanism through which flavonoids may reverse depressive-like behaviours in rodents via increasing circulating brain-derived neurotropic factor (BDNF).^18^ This effect has been further explored in a study by Neshatdoust et al. (2016)^19^ where participants that consumed a high flavonoid diet for 18-weeks had a significant increase in serum BDNF levels. The increase in BDNF in this study was further associated with improved cognitive function, though mood was not tested representing an interesting area to investigate whether maintaining neuroplasticity via BDNF regulation from flavonoid supplementation benefits mood.

Previous reviews of studies of the effects of flavonoids on psychological wellbeing^20–22^ have suggested possible benefits for depression, anxiety, and overall mood. However, the findings of these investigations also indicate that the evidence is hampered by methodological disparity, suggesting that more conclusive evidence for mechanistic pathways is needed. One important factor regarding the consideration of public health interventions is the accessibility of these beneficial phytochemicals. Accordingly, this review focussed solely on the benefits of flavonoids that can be consumed in the diet, either from a food or drink in its natural state, or in a freeze-dried form.

Therefore, the aim is to conduct a systematic review of human experimental studies investigating the efficacy of dietary flavonoids on mood and mental health in healthy populations across the lifespan.

## METHODS

This review followed the PRISMA 2020 guidelines (Preferred Reporting Items for Systematic Reviews and Meta-Analyses) and was registered in the International Prospective Register of Systematic Reviews PROSPERO (no. CRD42021293040).

### Eligibility Criteria

The eligibility criteria were defined according to the PICOS framework as shown in Table 1.

**Table 1.** PICOS framework.

Population	Human subjects, no specific age, gender, ethnicity. No restrictions on pre-existing mood or mental health conditions.
Intervention	Experimental studies with flavonoid-rich foods on mood and/or mental health.
Comparator	Experimental trials with a well-matched, low-flavonoid control group.
Outcome	Self-reported mood and mental health.
Study design	Randomized controlled studies regardless of duration or blinding procedures.

Experimental human trials with human participants of any age, sex, or ethnicity were eligible for inclusion if the participants were provided with supplementation with at least 1 flavonoid-rich food in the form of either lyophilized powder or whole food and underwent measurement of at least 1 mood or mental health outcome. No restrictions were placed on interventions with various types of flavonoid-rich food or length of supplementation. Additionally, no restrictions were placed on participants with existing mood and mental health conditions. However, in order to assess outcomes in physically healthy populations, studies were excluded if participants had existing conditions that affected physical health or caused hormonal imbalance. In addition, reports of studies that explored the effects of flavonoids on mood in participants experiencing menopause were also excluded owing to the hormonal influence on mood during the menopausal period, which could have confounded the interpretation of results for this population.

The experimental studies included in this review were required to have a well-matched (in terms of composition), low-flavonoid control group and to have controlled for caffeine intake either methodologically or statistically. Foods were defined as flavonoid rich according to the report by Neshatdoust et al. (2016),^19^ who defined low-flavonoid foods as those containing between 5 and 15 mg/100 g total flavonoids, a cutoff which has also been previously utilized and reported in the studies by Chong et al. (2013)^23^ and Macready et al. (2014).^24^ Therefore, studies were excluded if they utilized foods or beverages that contained <15 mg per 100 g/mL of flavonoid constituents.

In this systematic review, in order to be as inclusive as possible with regard to mood and mental health outcomes, mood was defined as “a state of mind characterized by emotional and psychological wellbeing”. This working definition included any symptoms relating to mood disorders at either clinical or subclinical levels. For the purpose of this research, items and data relating to social wellbeing, social belonging, and social engagement were excluded and items and data relating to mood evaluations of cognitive performance or mental fatigue were also excluded. Studies were excluded if they did not assess mood or mental health per this definition. Additionally, studies were excluded if they included administration of flavonoid extracts to participants, and due to the confounding effects of alcohol on mood, experimental studies of red wine were also excluded.

### Search Strategy

The electronic databases PubMed, Web of Science, and Scopus were searched with no restriction on publication start date to October 2024. To identify peer-reviewed, English language publications that met the eligibility criteria, reference lists of relevant studies, including review articles, were also manually checked. The United States Department of Agriculture (USDA) database was used to determine the flavonoid content of selected foods.^25^ Each database was systematically searched with the search term list provided in the Supplementary Material.

In PubMed, the search was run through “all fields,” including the title, abstract, key words, and Medical Subject Headings (MeSH), by using the advanced search feature. The advanced search feature was also used for Scopus, in which the search was run through the ‘search within’ toolbar to include the article title, abstract, and key words. The Core Collection from Web of Science was used so that it did not return a number of duplicate references from Medline; here, each term was searched using the ‘topic’ search fields, which included title, abstract, and keywords. Full text, human, and English filters were applied to all searches to help refine the large volume of unrelated articles. The bibliographic data were managed using EndNote X9.2 (Clarivate, Philadelphia, PA, United States). Initial searches, removal of duplicates, and screening of title and abstract were performed by R.C., following which the included articles were independently verified by D.L.

Titles of the 15 398 records identified were manually screened using EndNote by 1 reviewer (R.C.), followed by abstract review of eligible titles. Abstracts meeting the criteria were then processed for full-text screening. A second reviewer (D.L.) independently verified the included articles. Discrepancies were resolved through discussion with a third reviewer (K.B.). No automated tools or software were used during the screening process.

Studies selected for inclusion were assessed using the Evidence Analysis Manual Quality Criteria Checklist (QCC) from the Academy of Nutrition and Dietetics Evidence Analysis Library^®^26^^ for quality of methodology and risk of bias. This tool was chosen because it is specifically designed to assess nutrition and dietary intervention studies, allowing for a more targeted and relevant evaluation of the included trials. All articles were included regardless of methodological quality so that current limitations within the literature could be highlighted. Data extraction was conducted using the Academy of Nutrition and Dietetics Evidence Analysis Library^®^ Evidence Analysis Manual Data Extraction Template to extract key information, including study design, type of flavonoid-rich food, treatment duration, participant characteristics, outcome measures, and results. Many articles also reported the exploration of physical and biochemical outcome measures; however, only data relevant to psychological outcome measures were extracted. Studies selected for synthesis are presented in a table (Table 2) to display individual study characteristics.

**Table 2.** Key characteristics of experimental trials.

Food category	Trial type	Age category, y	Citation	Design	**Sample (Age mean ± SD)**	Flavonoid source	Intervention details		Duration	Outcomes	Findings
							Experimental	Placebo			
Berry fruits	Acute	7-18	Khalid et al.(2017) Study 2 ^27^ United Kingdom	Cross over (*n* = 52)	Healthy children (8.24 ± 0.96)	WBB	30 g freeze-dried WBB with 30 mL orange squash and 170 mL water (253 mg anthocyanins)	Placebo drink matched for volume, taste, appearance, and sugar content.	2 h	PANAS-C	Significant interaction effect where there was a significant increase in PA after consuming the WBB drink, not seen in placebo. No effect on NA.
			Khalid et al. (2017) Study 1 ^27^ United Kin**g**dom	Crossover (*n* = 21)	Healthy young adults (20.14 ± 1.01)	WBB	30 g freeze dried WBB with 30 mL orange squash and 220 mL water (253 mg anthocyanins)	Placebo drink matched for volume, taste, appearance, and sugar content.	2 h	PANAS	Significant interaction effect where there was a significant increase in PA after consuming WBB drink, not seen in placebo. No effect on NA.
		18-35	Velichkov et al. (2024) acute data United Kingdom^28^	Parallel groups (*n* = 60)	Emerging adults with self-reported symptoms of depression (20 ± 1.4)	WBB	22 g freeze-dried WBB mixed with water (121 mg anthocyanins)	Placebo drink matched for volume, taste, appearance and sugar content.	2 h	PANAS-X	Significant effect of treatment, where PA scores are higher postintervention in low consumers of fruit and vegetables
			Haskell-Ramsay et al. (2017)^54^ United Kingdom	Crossover (*n* = 20)	Healthy young adults (21.05 ± 0.89)	Purple grape juice	200 mL commercially available purple grape juice plus 30 mL blackcurrant flavor cordial (138.3 mg/L anthocyanins)	200 mL commercially available white grape juice plus 10 mL blackcurrant flavor cordial and 20 mL cold water.	20 min	Bond–Lader VAS	Main effect of treatment found for calm ratings, where higher calm scores were found following consumption of purple grape compared to placebo.
			Whyte et al. (2019)^63^ United Kingdom	Parallel groups (*n* = 40)	Healthy young adults (22.8 ± 2.63)	Mixed berry	400 mL smoothie consisting of 75 g each of whole strawberries, blueberries, blackberries, and raspberries, blended with 100 mL water and containing 569.7 estimated flavonoid content.	Placebo drink matched for volume, taste, appearance, and sugar content.	2, 4, 6 h	PANAS-NOW	No effect of intervention on PA or NA.
	Chronic	7-18	Barfoot et al. (2021)a United Kingdom	Parallel groups (*n* = 15)	Healthy children (7-10-y-olds), (8.38 ± 0.93)	Wild blueberry (WBB)	WBB drink containing 253 mg anthocyanins prepared by mixing 170 mL water, 13.3 g freeze- dried WBB and 30 mL of low-flavonoid Rocks Orange Squash.	Placebo beverage matched for sugar content taste and color	4 wk (with a 2 wk visit)	PANAS-C	PA lower at 4 wk compared to 2 wk, no significant effect of intervention drinks on PA or NA.
			Fisk et al. (2020)^42^ United Kingdom	Parallel groups (*n* = 64)	Healthy adolescents (14.20 ± 1.71)	WBB	13 g of freeze-dried WBB powder (containing about 253 mg anthocyanins)	Placebo beverage matched for sugar content, taste and color.	4- wk, 2-week checkpoint where PANAS-NOW completed.	MFQ, RCADS, PANAS-NOW	MFQ scores were significantly lower at 4 wk when consuming WBB compared to placebo. No effect on RCADS or PANAS at 2 and 4 wk
		18-35	Velichkov et al. (2024)^28^ chronic data United Kingdom	Parallel groups (*n* = 60)	Emerging adults with self-reported symptoms of depression (20 ± 1.4)	WBB	22g freeze dried WBB mixed with water (121 mg anthocyanins)	Placebo drink matched for volume, taste, appearance and sugar content.	6-wk	BDI, PANAS-X, PHQ-9, GAD-7, SHAPS, PSS-10	No effects of intervention
			Sinclair et al. (2022)^62^ United Kingdom	Parallel groups (*n* = 44)	Healthy adults (34 ± 13)	Blueberry and cherry	(1) 60 mL blueberry concentrate diluted in water (774 mg anthocyanins) (2) 60 mL tart cherry concentrate diluted in water (640 mg anthocyanins)	Placebo beverage matched for sugar content, flavor and color	20 days	BDI-II, STAI	Significant interaction effect where depression and anxiety scores significantly reduced following blueberry treatment compared to placebo. Anxiety scores also reduced significantly in blueberry arm compared to cherry.
		35-65	Lamport et al. (2016)^65^ United Kingdom	Cross over (*n* = 19)	Healthy mothers of preteen children (42.8 ± 0.7)	Concord grape juice	355 mL concord grape juice/d (777 mg polyphenolics as a gallic acid equivalent/355-mL daily serving (167 mg anthocyanins as malvidin equivalent and 334 mg proanthocyanidins as catechin equivalent).	Placebo beverage matched for sugar content, taste and color	12- wk	Bond-Lader VAS, STAI-6	No significant interaction effects of treatment, some treatment and study phase interactions seen for Bond-Lader scores
			Kimble et al. (2022) United Kingdom	Parallel groups (*n* = 50)	Non-smoking adults, low consumers of fruit and vegetables, low levels of physical activity with a risk factor for type II diabetes (48 ± 6)	Montmorency cherries	30 mL cherry juice found to contain 370·2 (sd: 112·2) mg/L of cyanidin-3-glucoside equivalents and 3259·0 (sd: 218·9) mg/L gallic acid equivalents diluted in ∼240 mL water.	Placebo beverage matched for sugar content, taste and colour	3-months	Bond-Lader VAS	Main effect of treatment where alertness was higher in the intervention group. Post supplementation, mental fatigue was lower in the intervention group.
			Krikorian et al. (2022)^61^ United States	Parallel groups (*n* = 27)	Middle aged overweight adults (56.4 ± ns)	WBB	12 g freeze-dried blueberries/d mixed with water, flavonoid content not reported	Placebo powder matched for sugar content, flavour and color	12 wk	BDI-II	No significant effect of Intervention
			Garrido et al. (2012)^30^ Spain	Cross over (*n* = 30)	Healthy volunteers, mixed age range 20-30, 35-55, 65-85	Jerte Valley cherry	27.85g cherry product consisting of 18.85 g pitted freeze-dried cherries (equivalent to 141 g fresh cherries) plus 7.5 g maltodextrin and 1.5 g ascorbic acid dried to a powder then diluted in water producing 125 mL cherry based product per dose. 1 dose of the product provided roughly 1580 mg phenolic compounds (expressed as gallic acid equivalents), 30 mg anthocyanins (calculated as malvidin equivalents)	Commercial cherry flavoured soft drink.	5 days (consumed twice a day)	STAI	Significant main effect of intervention on state anxiety for middle-aged and elderly participants compared to baseline. Effect was maintained 1-day after the intervention was discontinued. A significant decrease in trait anxiety in middle-aged and elderly, compared to baseline, effect also maintained 1-day post-trial. No changes found as a result of consuming placebo.
		65+	Miller et al. (2018)^60^ United States	Parallel groups (*n* = 37)	Healthy older adults (67.6 ± 4.7)	WBB	24 g freeze-dried blueberries/d mixed with water (461 mg anthocyanins)	Placebo powder matched for sugar content, flavour and aroma	3 months	GDS, POMS	No significant effect of intervention on mood outcomes
			Miller et al. (2021)^59^ United States	Parallel groups (*n* = 37)	Healthy older adults (67.6 ± 4.3 years)	Strawberries	24 g freeze-dried strawberries/d mixed with water, 114 mg flavonoids ^88^	Placebo powder matched for sugar content, flavour and aroma	3 months	GDS, POMS	No significant effect of intervention.
			Crews et al. (2005)^51^ United States	Parallel groups (*n* = 50)	Healthy older adults (69.28 ± 6.45)	Cranberry juice	Low calorie cranberry juice product containing 27% juice/volume, flavonoid content not reported	Placebo beverage matched for sugar content, taste and color	6-wk	Follow up self-report questionnaire measuring perceptions of participants moods	No significant effects of treatment.
Cocoa	Acute	18-35	Lamport et al., 2020)^70^ United Kingdom	Parallel groups (*n* = 98)	Healthy young adults, (20.65 ± 0.18)	Cocoa	35g commercially available dark chocolate bar (70% cocoa) 83 mg flavonoids.	35 g commercially available white chocolate bar	2hr	PANAS, Bond-Lader VAS	No effects of treatment for any outcomes.
			Boolani et al. (2017)^53^ United States	Cross over (*n* = 23)	Low polyphenol consumers without elevated feelings of energy (20.25 ± 2.23)	Cocoa	473 mL cocoa drink 499 mg flavanols	473 mL placebo drink matched for volume, taste, appearance and sugar content.	22-48 mins, 60-86 mins, 98-124 mins	POMS	No effects of treatment for any outcomes.
			Scholey et al. (2010)^36^ Uni**t**ed Kingdom	Cross over (*n* = 30)	Healthy student volunteers (21.9 ± 0.61)	Cocoa	Two doses of cocoa powder mixed with 200 mL hot water. Powders contained either 520 mg cocoa flavanols or 994 mg cocoa flavanols.	Cocoa powder containing 46 mg CF mixed with 200 mL hot water.	90 min	STAI	No effect of intervention on STAI.
			Martin et al. (2012)^50^ The Netherlands	Parallel groups (*n* = 90)	Healthy participants, divided into high and low anxiety, according to baseline STAI scores (22.8 ± 3.6)	Cocoa	20 g dark chocolate (75%) 25 g milk chocolate KitKat, flavonoid content not reported	2 crackers and 15 g cheese spread	60 min	STAI	High trait anxiety individuals had higher state anxiety 60 mins after eating the milk chocolate bar The cheese and cracker group had lower state anxiety at 60 mins and no changes were found in the dark chocolate group or for the low trait anxiety group for any product.
		35-65	Pase et al. (2013)^29^ (acute data) Australia	Parallel groups (*n* = 71)	Healthy middle-aged adults (52.37 ± 7.72)	Cocoa	20g dark chocolate drink mix containing either 250 mg or 500 mg cocoa flavonols in 200 mL water	20g dark chocolate drink mix containing 0 polyphenols in 200 mL water	1, 2.5, 4 hr	Bond-Lader VAS	No effect of intervention on any outcomes.
			Marsh et al. (2017)^47^ Australia	Cross over (*n* = 14)	Healthy postmenopausal women (57.6 ± 4.8)	Cocoa	84 g of a high concentration cocoa (80%) ‘dark’ chocolate (395 mg polyphenols), or 87 g of a lower concentration cocoa (35%) ‘milk’ chocolate (200 mg polyphenols)	85 g of a cocoa butter ‘white’ chocolate (0% cocoa solids) (35 mg polyphenols)	30, 90 mins	POMS-A	No effect of intervention
	Chronic	18-35	Shin et al. (2022)^58^ South Korea	Parallel groups (*n* = 48)	Healthy adults (23.95 ± 3.05)	Cocoa	10g of 1. 85% cocoa chocolate 2. 70% cocoa chocolate 3 times a day, flavonoid content not reported	Not supplied any chocolate	3 wk	PANAS	Significant effect of treatment where negative affect scores were significantly reduced following consumption of 85% dark chocolate
		35-65	Tsang et al. (2019)^57^ United Kingdom	Parallel groups (*n* = 26)	Healthy adults (38.8 ± 11.1)	Cocoa	25 g serving of high polyphenol dark chocolate (HPDC) which contained 500 mg flavonoids	Similar serving of low polyphenol dark chocolate (LPDC) containing negligible flavonoids.	4-wk	PANAS	No significant interaction effect for PA or NA. Within groups treatment effect was seen where a significant main effect of time and treatment on NA scores following high polyphenol dark chocolate, not seen in low polyphenol group.
			Pase et al. (2013) ^29^ (chronic data) Australia	Parallel groups (*n* = 71)	Healthy middle-aged adults (52.37 ± 7.72)	Cocoa	20g dark chocolate drink mix containing either 1) 250 mg or 2) 500 mg cocoa flavonols in 200 mL water	20g dark chocolate drink mix containing 0 polyphenols in 200 mL water	30-days	Bond-Lader VAS	Significant effect of treatment for Calm and Content VAS. Significant increase found for calmness and contentedness in high polyphenol group but not for low or placebo.
Orange juice	Acute	35-65	Alharbi et al. (2016)^89^ United Kingdom	Cross over (*n* = 24)	Healthy males (51 ± 6.6)	Orange juice	240- mL flavonoid rich orange juice (272 mg flavonoids).	240- mL placebo drink matched for volume, taste, appearance, energy and sugar content.	Acute 2, 6 hours	PANAS	Main effect of drink for ‘alertness’ subscale, no other significant main effects or interactions.
	Chronic	18-35	Park et al. (2020)^79^ South Korea	Parallel groups (*n* = 40)	Young adults (21.83 ± 2.43)	Orange juice	300 mL orange juice (600 mg flavonoids)	300 mL matched placebo	8 wk	CES-D	Significant reduction in CES-D scores in intervention group not seen in placebo.
		65+	Kean et al., 2015)^39^ United Kingdom	Cross over (*n* = 37)	Healthy older adults (66.7 ± 5.3)	Orange juice	500mL serving of orange juice containing 305 mg flavanones	500mL matched placebo drink containing 37 mg flavanones	8 wk	PANAS	No significant effect of intervention
Green tea	Chronic	18-35	Zhang et al. (2013)^56^ China	Parallel groups (*n* = 46)	Healthy participants (25.67 ± 4.61)	Green tea	400mg 3 times a day	Cellulose 3 times a day	5 wk	MADRS, HRSD-17	Significant decrease in scores for MADRS and HRSD-17 in green tea group, not observed in placebo
Soy	Chronic	35-55	Simpson et al. (2019)^77^ United Kingdom	Parallel groups (*n* = 101)	Postmenopausal women (53.75 ± 3.87)	Soy	1. Medium (35 mg/350 mL), 2. or high (60 mg/600 mL) dose of isoflavones contained within a soy drink	Low dose isoflavones within a soy drink (10 mg/100 mL)	12 wk	PANAS	Significant difference between doses but no significant interaction for PA or NA.
		65+	Kok et al. (2005)^55^ The Netherlands	Parallel groups (*n* = 202)	Postmenopausal women (66.7 ± 4.75)	Soy	36.5 g of powder containing 25.6 g soy protein containing 52 mg genistein, 41 mg daidzein, and 6 mg glycitein aglycone weights as a powder.	Placebo powder matched for taste and appearance.	12 months	SF-36 mental health dimension, GDS	No significant interaction for any mood outcome
Tree nuts	Chronic	18-35	Pribis (2016)^44^ United States	Cross over (*n* = 64)	Young adults (20.65 ± 2.05)	Walnuts	60g English walnuts in banana bread, flavonoid content not reported	Banana bread without walnuts	8 wk	POMS	No significant effect of intervention.
			Herselman et al. (2022)^84^ Australia	Parallel groups (*n* = 60)	Healthy undergraduate university students during examination period (22.0 ± ns)	Walnuts	56 g walnuts/d, flavonoid content not reported	Continue normal diet, avoid nuts or fatty fish for study duration.	16 wk	DASS21, MHC, POMS	Trend for improvement in depressive symptoms on the DASS21 in walnut group where participants did not have an increase in depression over exam period as seen in control group, though not a significant interaction. Similar results observed in the Psychological well-being
		65+	Coates et al. (2020)^69^ Australia	Parallel groups (*n* = 128)	Postmenopausal women (65 ± 8)	Almonds	Based on participants individual estimated energy requirements (EER), participants were provided with a portion of snack foods equivalent to ∼15% of their EER.	Carbohydrate rich snack foods (The Original Scotch Finger, Arnott’s Biscuits, North Strathfield, Australia and No Added Salt Potato Chips, Freedom Foods, Taren Point, Australia).	6 days per week for 12 wk	Bond-Lader VAS, POMS	No significant effects of treatment for any mood outcome.
Apples	Chronic	35-65	Bondonno et al. (2014) Australia	Cross over (*n* = 30)	Healthy middle-aged adults (47.3 ± 13.6)	Apples	Prepared by homogenising Cripps Pink apple skin (80 g) and apple flesh (120 g), where, half of each dose was provided raw and the other half was provided cooked (184 mg of total quercetin glycosides and 180 mg of (−)-epicatechin).	Apple flesh only (less than 5 mg of total quercetin glycosides and (−)-epicatechin).	Acute 2.5 hours	Bond-Lader VAS	No significant effect of treatment found.
Peppermint	Chronic	18-35	Abdelhalim (2021)^49^ Saudi Arabia	Parallel groups (*n* = 124)	Healthy young adults (21.96 ± 1.7)	Peppermint	Infusion of 250 mg fresh aerial parts of the peppermint plant (infused for 10 min in hot water) 30 min before bedtime daily for 30 days, flavonoid content not reported	Asked not to drink peppermint or any other herbs	30 days	STAI	Significant reduction in STAI scores in intervention group.
Mixed foods	Chronic	18-35	Barfoot et al. (2021) b^52^ United Kingdom	Parallel groups (*n* = 38)	Postnatal mothers (1 year), (29.21 ± 5.67)	Mixed	Added 1 high flavonoid food item/d to current diet including ‘berry fruits (∼120 g) e.g. blueberries, raspberries, strawberries, blackberries, blackcurrants, mixed berries’, ‘2 large squares of (min. 70% cocoa) dark chocolate’, ‘4–5 cups of black/green tea or caffeinated/decaffeinated coffee’, ‘1 large glass of red wine (250 mL)’, ‘1 portion of leafy green vegetables such as spinach or cabbage (∼70 g)’ and ‘1 glass (250 mL) of fresh orange or grapefruit juice (not from concentrate)’.	Continue normal diet	14-days	STAI, PHQ-8, PANAS , WHOQOL-BREF	Significant interaction effect for state anxiety where mothers in the experimental group reported lower state anxiety after the intervention compared to controls.
			Colombage et al. (2024)^78^ United Kingdom	Parallel groups (*n* = 38)	Postnatal mothers (6 months), (35.11 ± 3.77)	Mixed	Added 2 high flavonoid food item/d to current diet including berry fruit (∼120 g) e.g. blueberries, raspberries, strawberries, blackberries, blackcurrants, mixed berries’, ‘2 large squares of (min. 70% cocoa) dark chocolate’, ‘4–5 cups of black/green tea or caffeinated/decaffeinated coffee’, ‘1 large glass of red wine (250 mL)’, ‘1 portion of leafy green vegetables such as spinach or cabbage (∼70 g)’ and ‘1 glass (250 mL) of fresh orange or grapefruit juice (not from concentrate)’.	Continue normal diet	14-days	STAI, EPDS, PANAS, WHOQOL-BREF, PSAS-RSF-C	Significant interaction effect for postnatal depression and positive affect, where participants in the experimental condition reported lower EPDS scores and higher PA scores following the intervention.

## RESULTS

### Study Characteristics

**Figure 1.**
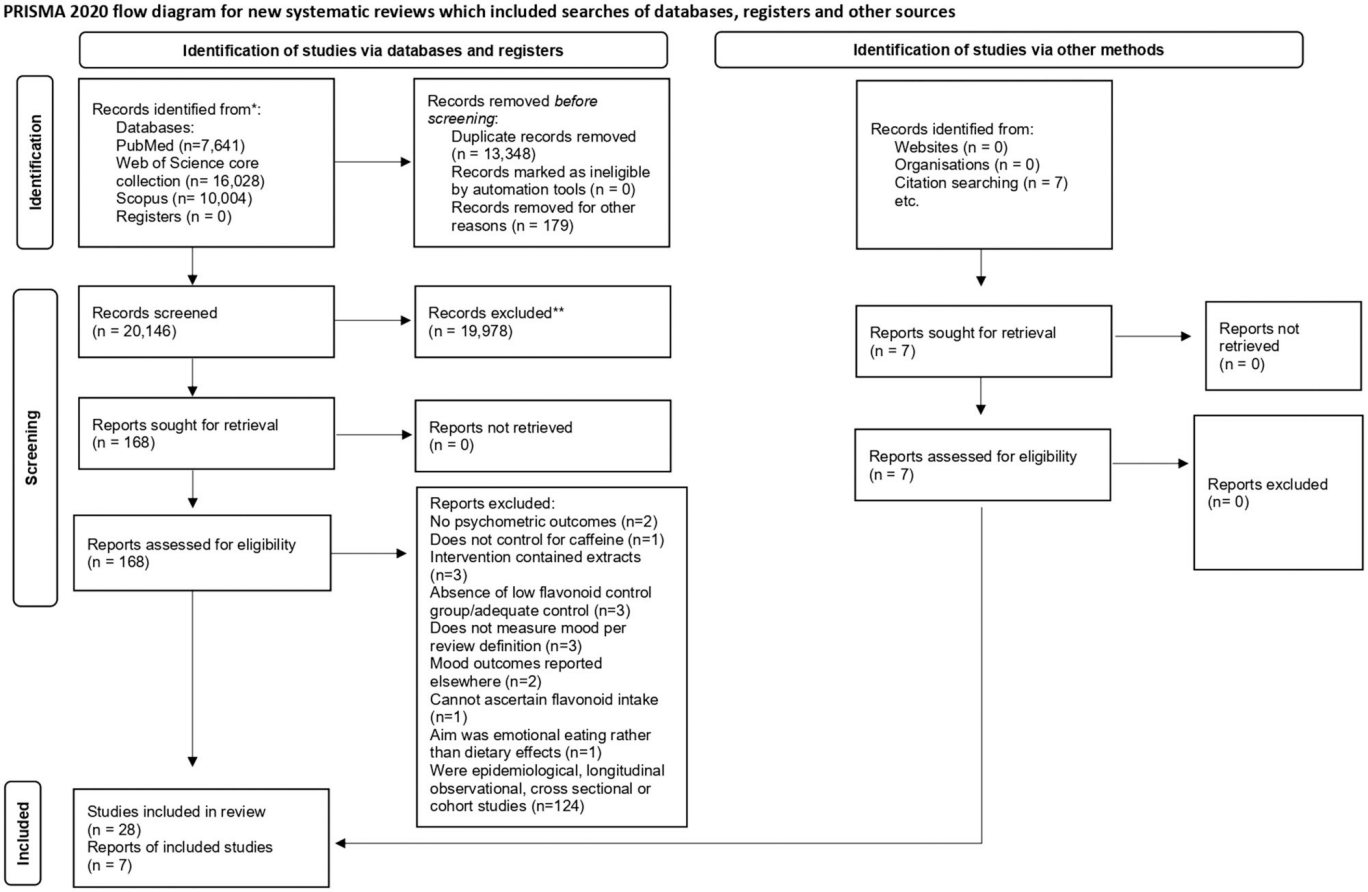
Preferred Reporting Items for Systematic Reviews and Meta-Analyses (PRISMA) flow diagram for the identification of studies included in the review.

This search identified 35 publications (Figure 1); however, the article by Khalid et al. (2017)^27^ reported outcomes from 2 studies by Velichkov et al. (2024)^28^ and Pase et al. (2013),^29^ who reported acute and chronic outcomes. As such, 38 datasets are reported, of which 13 were acute and 25 were chronic. It is worth noting that for the age group subcategory there was a total of 40 studies because of the intention to analyze each age category in Garrido et al. (2012)^30^ as separate studies. The review inclusion and exclusion criteria reported in the study are reported in the PRISMA flow diagram: 2 studies did not have psychometric outcomes,^31^^,^^32^ 1 intervention contained caffeine,^33^ 3 studies used flavonoid extracts,^34–36^ 3 studies did not have an adequate low-flavonoid control group,^37^^,^^38^ 3 studies did not measure mood per our definition,^39–41^ 2 studies reported mood outcomes elsewhere,^44^^,^^68^ 1 study could not ascertain exact flavonoid content in the intervention,^45^ and 1 study focused on emotional eating.^46^

The interventions included in the eligible studies consisted of wild blueberry (WBB; *n *= 8), cocoa (*n *= 9), grape (*n *= 2), berry (*n *= 6), green tea (*n *= 1), orange juice (*n *= 3), soy (*n *= 2), nuts (*n *= 32), apples (*n *= 1), peppermint (*n *= 1), and a range of flavonoid foods *(n *= 2). The most commonly used intervention was cocoa, for which flavonoid doses ranged from 16.72^47^ to 994 mg.^48^ For cases in which studies did not state the exact flavonoid content, databases were used (http://phenol-explorer.eu/contents/food/439 and the USDA) to calculate flavonoid content based on the weight of the intervention given.^47^^,^^49^^,^^50^ Twenty-one of 25 chronic studies and 4 of 13 acute studies used parallel groups design. The duration of the chronic studies ranged from 5 days to 12 months. Included studies were conducted in 1972 participants with ages ranging from 8^27^ to 69 years^51^ and with the majority (*n = *17) of studies performed in young adult populations (20-35 years old). The most commonly used measures in both acute and chronic designs were the Positive and Negative Affect Schedule (*n* = 6 acute, *n *= 8 chronic), followed by the Profile of Mood States in Chronic Designs (*n* = 5), and the Bond–Lader Visual Analogue Scale (VAS) in acute designs (*n* =*4*).

### Methodological Quality

Using the QCC, we rated 8 studies as “positive,” indicating less risk of bias,^28^^,^^47^^,^^52–55^^,^^64^^,^^65^^,^^77^^,^^78^ and 3 studies as “negative”^49^^,^^50^^,^^79^ because of frequent lack of reporting of withdrawals or use of inappropriate blinding procedures. The remaining studies were rated as “neutral,” indicating a low risk of bias from funding sources, outcome measures, and regimen. However, randomization of participants, reporting of withdrawals, and stating of clear research questions presented a lower risk of bias.

### Findings From Acute Studies

Of the 13 studies included, 5 studies (38%) found evidence that acute flavonoid supplementation with anthocyanin-rich foods held benefits for mood. Khalid et al. (2017)^27^ (studies 1 and 2) found improved mood, measured with the Positive and Negative Affect Schedule in both child and young adult populations 2 hours following a wild blueberry (WBB) intervention (253 mg anthocyanins). Additionally, Haskell-Ramsay et al. (2017)^54^ found an improvement in calm ratings in healthy young adults by using the Bond–Lader VAS 20 minutes following a purple grape juice intervention (138.3 mg/L anthocyanins). Finally, Velichkov et al. (2024)^28^ found a main effect of treatment with WBB, whereby the positive affect of study participants increased in the experimental condition 2 hours after consumption in those with low fruit and vegetable intake.

Considering the acute literature, it is worth noting that in the study by Haskell-Ramsay et al. (2017)^54^ 20 minutes may have been too short a time period to elicit the benefits of flavonoids, given that circulating anthocyanin metabolites peak between 1-2 and 6 hours (Rodriguez-Mateos et al., 2013)^80^ post-consumption. Therefore, where effects are observed in a timeframe of <1 hour, it is important to consider non–flavonoid-related mechanisms when interpreting findings. On the other hand, cocoa was used as an intervention vehicle for 50% of the included acute studies, none of which found positive effects on mood. However, improvements in alertness were also found 2 hours following consumption of flavonoid-rich orange juice, supporting that orange juice may also support mood acutely (Alharbi et al. [2016]^89^). Overall, these results suggest that anthocyanin-based interventions, with anthocyanin doses between 183 and 253 mg, in addition to 272 mg flavonoids in orange juice and with outcomes measured approximately 2 hours following the intervention, may be optimal doses and timepoints for observing acute benefits of flavonoids on mood. Other flavonoid-rich foods may be less promising for acute effects on mood.

### Findings From Chronic Studies

Of the 25 chronic studies included, 12 (48%) found a significant effect of flavonoids on mood and/or mental health. Due to the diversity of the interventions used, there was not clear evidence for any singular food being most likely to confer benefits if consumed over a sustained period. However, 3 of 4 studies that utilized the State Trait Anxiety Inventory (STAI) found benefits of flavonoids on anxiety symptoms, highlighting a potentially sensitive measure to detect mood changes in chronic interventions. For example, Garrido et al. (2012)^30^ conducted a crossover study utilizing Jerte Valley cherries over a period of 5 days and found significant improvements in anxiety using the STAI. This finding was also reported by Sinclair et al. (2022)^62^ and Barfoot et al. (2021 b),^52^ who found improvements in anxiety symptoms (with the STAI) following a flavonoid intervention with blueberries for a period of 20 days and mixed-flavonoid rich foods for 14 days, respectively. Furthermore, 9 of the included studies that did not find effects were conducted in middle-age and older adults, which may indicate that individuals in this population are less likely to benefit from flavonoid interventions, particularly in studies with chronic designs.

### Flavonoid Food Sources

#### Blueberry

Seven studies used a WBB intervention, 4 (57%) of these studies found benefits of flavonoids. Khalid et al. (2017)^27^ found a significant increase in positive affect following a WBB intervention at 2 hours post-consumption in both children and young adults. Fisk et al. (2020)^42^ also found benefits to mood in healthy adolescents, who had a significant decrease in scores on the Mood and Feelings Questionnaire following a 4 week WBB intervention, representing a reduction in depression symptoms in this cohort. In these 3 datasets all interventions used a 253 mg dose of anthocyanin (equivalent to 240 g of fresh blueberries), representing a potentially optimal dose to elicit changes in mood. On the other hand, Velichkov et al. (2024)^28^ used a smaller dose of anthocyanins (121 mg) and found an increase in positive affect. This finding was present in a population with self-reported depressive symptoms, which, combined with the smaller dose (150 vs 240 g fresh fruit equivalent in the other acute WBB trials), promotes the potential for acute WBB interventions for clinical as well as general populations for mood and mental health. Comparatively, all of the studies that did not find significant effects had chronic designs using a range of durations from 4 weeks to 3 months. For example, Miller et al. (2018)^59^ did not find mood effects following a 3 month blueberry intervention (19.2 mg anthocyanins) in healthy older adults; in addition, wild blueberries (253 mg anthocyanins) did not elicit changes in mood in children 7-10 years old following a 4 week intervention (Barfoot et al. 2021a).^43^ However, it is worth noting that this particular study ran a post-hoc power analysis on the cognitive outcome findings that revealed low power, which therefore may affect outcomes. Overall, these findings may indicate that acute doses of blueberries may lead to better mood outcomes than consumption over a period of time, while also suggesting that adolescents and young adults may benefit most from these interventions.

#### Cocoa

Three of the 9 studies (33%) that investigated cocoa as an intervention found significant effects of supplementation on mood. The studies finding benefits of the intervention were all chronic studies, with 2 studies lasting 30 days and 1 study lasting 4 weeks. Pase et al. (2013)^29^ found that healthy middle-aged adults given supplementation with a 20-g dark chocolate drink (500 mg cocoa flavonols) had a significant increase in calmness and contentedness after 30 days, an effect that was not replicated in the low-flavonol group (250 mg cocoa flavonols) or the control group. Similarly, Tsang et al. (2019)^57^ found significant within-group treatment effects for negative affect following 500-mg cocoa flavonoid supplementation for 4 weeks in adults, a finding that indicates a decrease in negative mood following the intervention. The remaining studies finding non-significant effects of cocoa flavonoids on mood were all conducted in an acute setting. Together, these study results suggest an optimal supplementation duration of 4 weeks and dosage of around 500 mg/d of flavonols for cocoa interventions on mood.

#### Grape

Only 2 studies in this review used grape as an intervention. One of these studies (50%; Haskell-Ramsay et al. [2017]^54^) found positive effects on mood following supplementation. In comparison, Lamport et al. (2016)^65^ conducted a 12-week intervention in mothers of preteen children using concord grape juice (167 mg anthocyanins as the malvidin equivalent and 334 mg proanthocyanidins as the catechin equivalent). These studies both used the Bond–Lader VAS; however, Lamport et al. (2016)^65^ did not find significant effects such as those seen in the study by Haskell-Ramsay et al. (2017).^54^ These findings could be partly explained by the study population, whereby the mothers of preteen children were likely to have been maximally stressed, which was a purposeful aim of the study; therefore, the administration of concord grape juice in the Lamport et al. study population was not as effective as administration to the healthy young adult population in the Haskell-Ramsay et al. (2017)^54^ study. Furthermore, differences can also be noted in the total phenolic concentration of the active treatment groups, which according to the article by Haskell-Ramsay et al. (2017)^54^ had a total phenolic content of 336.34 mg gallic acid equivalent in the 200 mL intervention, whereas Lamport et al. (2016)^65^ reported 777 mg total polyphenolics as a gallic acid equivalent in the 355 mL intervention. Therefore, it could be suggested that the administration of larger quantities of total phenolics may not confer greater benefits to mood than smaller quantities.

#### Berry (Not Including Blueberry)

Six studies used a berry intervention (cherry, strawberry, cranberry, mixed berry, and cherry), of which 3 studies (50%) found significant effects on mood using the STAI, BDI-II, and the Bond-Lader alertness scale. Of the trials that found effects, all were chronic studies lasting between 5 days - 3 months. Comparatively, of the 3 studies that did not find effects, 1 used an acute design (Whyte et al. 2019)^63^ and 2 used chronic designs (Crews et al. 2005;^51^ Miller et al. 2021^59^). Miller et al. (2021)^59^ conducted a parallel groups design study over a 3 month period, in which supplementation with 24 g freeze-dried strawberries was given to older adults and did not find effects on results of the Geriatric Depression Scale (GDS) or the Profile of Mood States (POMS), which measures a range of mood states including anxiety, anger, fatigue, and depression. Kimble et al. (2022),^76^ on the other hand, found that supplementing participants with Montmorency cherries for 3 months led to improvements in alertness following the intervention. Although the change in alertness is only 1 facet of 3 from the outcome measure that was sensitive to the intervention, this finding is interesting considering the study population, in which participants were low consumers of fruit and vegetables and had low levels of physical activity with a risk factor for type 2 diabetes. This finding has occurred consistently in findings for recent research, whereby consumers of low amounts of fruit and vegetables seemed to benefit from a dietary intervention in terms of mood and wellbeing (eg, Conner et al. 2017^11^ and Kontogianni et al. 2020^12^). Recently, Velichkov et al. (2024)^28^ found that participants following 6 weeks of WBB supplementation who consumed <3 portions of fruit and vegetables per week had improvements in positive affect to a greater extent than participants who were high consumers of fruit and vegetables per week. Collectively, these results may signify that benefits of flavonoid interventions may be more prominent in specific populations, such as those with low levels of fruit and vegetable intake or physical activity participation.

#### Green Tea

There was only 1 included study that explored the effects of green tea on mood. Zhang et al. (2013)^56^ conducted a parallel groups trial, supplementing healthy young adults with green tea 3 times a day for 5 weeks. This trial utilized the Montgomery–Åsberg Depression Rating Scale and the Hamilton Rating Scale for Depression, 17-item, both measuring depression, and found a significant decrease in scores in the green tea group that was not observed in the cellulose-supplemented placebo group. These results indicate that green tea may be effective in reducing depression scores in this cohort.

#### Orange Juice

Two of the 3 (66%) studies evaluating the effects of orange juice on mood found significant benefits to mental health following the intervention. Park et al. (2020)^79^ supplemented young adults for 8 weeks with 300 mL orange juice and found improvements in scores of the Center for Epidemiologic Studies Depression Scale (CES-D) following the intervention. Additionally, a main effect of treatment was found in the article by Alharbi et al. (2016),^89^ whereby the flavonoid-rich drink attenuated a decline in alertness seen in the placebo at 2 hours. In contrast, no significant changes in mood were seen during another 8 week intervention (Kean et al. 2015).^64^ It is noteworthy, however, that the disparity in findings between Kean et al. (2015)^64^ and Park et al. (2020)^79^ may have been due to the lower quantity of flavanones in the Kean et al. (2015)^64^ study (305 mg) compared to nearly double (600 mg) in the later trial by Park et al., or perhaps the different study populations from young adults to older adults, in which younger adults may have benefitted more from the intervention compared to older adults. Interestingly, both Kean et al. (2015)^64^ and Alharbi et al. (2016)^89^ utilized the Positive and Negative Affect Schedule (PANAS) in their work, which was found to be sensitive to other food interventions such as berries, as mentioned above, suggesting an ideal measure of transient mood for nutritional interventions. However, Park et al. (2020)^79^ used the CES-D, which detected changes in mood, indicating this measure may also be sensitive to flavonoid supplementation. In reviews of the studies of dietary flavonoids for mood, older adults seemed to benefit less from the intervention, as seen in Crews et al. (2005);^51^ Kok et al. (2005);^55^ Miller et al. (2018)^60^ and Miller et al. (2021),^59^ all of which had study durations of 6-12 weeks, similar to those of the study by Kean et al. (2015).^64^ Therefore, although the higher flavanone content may explain the disparity in findings in the orange juice subclass, it may be that overall mood and mental health of older adults may be less sensitive to dietary flavonoid interventions than that of other age groups.

#### Soy

In regard to soy studies, 2 chronic studies by Kok et al. (2005)^55^ and Simpson et al. (2019)^77^ did not find benefits to mood and mental health as a result of a 12 month and 12 week supplementation period, respectively. Both studies used a similar study design, with the same population of postmenopausal women, highlighting that finding that chronic supplementation of soy may not elicit mood benefits in this population. It could be said, however, that mood is much more stable in postmenopausal women in comparison to those who are peri-menopausal or going through the menopause transition period (Brown et al. 2015).^75^ Therefore, these studies may have missed the most sensitive period for mood change in this population.

#### Tree nuts

None of the included studies showed a benefit of tree nut intervention on mood and mental health. All studies evaluated the intervention in a chronic design ranging from 8 to 16 weeks. The POMS tool was used in all interventions, highlighting the finding that this measure may not be particularly sensitive for detecting mood effects following flavonoid supplementation. Furthermore, there could be a need to develop more standardized protocols for control groups in nutritional interventions, whereby Herselman et al. (2022)^84^ asked participants in their control group to continue with their normal diet, and to avoid other nuts and fatty fish for the study duration. The results of this assessment indicated that a potential withdrawal effect may be present within this study and highlighted the need for further research of flavonoid-rich tree nuts on mood and mental health outcomes with more standardized procedures and better use of nutritionally matched placebos.

#### Apples

One study (Bondonno et al. 2014)^74^ explored the acute effects of apples on mood using the Bond–Lader VAS. This was a crossover trial with apple skin and apple flesh, providing 184 mg of total quercetin glycosides and 180 mg of (−)-epicatechin. Mood was tested at 2.5 hours and no benefits of the apple intervention were observed.

#### Peppermint

Abdelhalim (2021)^49^ supplemented healthy young adults with 250 mg peppermint tea (estimated total flavonoids 147 mg) for a period of 30 days and found that participants had a significant reduction of state anxiety (STAI) scores postintervention compared to those in the control group.

#### Range of Foods

Barfoot et al. (2021b)^52^ evaluated mood after an intervention of a range of flavonoid-rich foods. These authors asked a sample of postnatal mothers (0-12 months postpartum) to include a single flavonoid rich food into their diet for a period of 2 weeks. After the intervention, Barfoot et al. found that the mothers had a significant reduction in state anxiety (STAI) in the intervention group that was not seen in the control group. In a similar study, Colombage et al. (2024)^78^ found a significant decrease in postnatal depression scores and increase in positive affect in postnatal mothers (0-6 months postpartum) after consuming 2 flavonoid-rich foods in their diet every day for 2 weeks. Overall, these findings show promise for the effects of flavonoid supplementation on a range of mood outcomes during the postpartum period.

### Findings by Age Groups

For this analysis, there is a total of 37 studies, as it was intended to analyze each age category by Garrido et al. (2012)^30^ as separate studies.

#### Children and Adolescents (age 7-18 years)

Three studies included in this review used a sample of children and adolescents (7-18 years old). Only 1 of the studies did not find significant benefits from the flavonoid intervention (Barfoot et al. 2021a).^43^ All trials used the same flavonoid content (253 mg anthocyanins) with similar designs and outcome measures (PANAS used in Fisk et al. [2020];^42^ PANAS-C [PANAS in Children] in Barfoot et al. [2021a]^43^). Simple comparison of these studies indicated that adolescents may be more sensitive than children to mood changes as a result of a chronic flavonoid-rich blueberry intervention; however, it is worth noting that there are no studies in these populations with other flavonoid-rich foods. Interestingly, these findings may be strengthened due to the fact that adolescence is a particularly sensitive period for development, whereby there are changes in emotion and executive functioning from prefrontal cortex growth (Ahmed et al. 2015;^73^ Best and Miller, 2010;^67^ Davey et al. 2008;^66^ De Luca et al. 2003^72^); therefore, the effects of flavonoids could be enhanced during this period of development.

#### Young Adults (19-35 years old)

This age category was the largest, including 17 trials, of which 8 studies (47%) were found to show benefits of flavonoids on mood. Most of these studies were chronic studies, ranging from 5 days to 16 weeks, utilizing a range of flavonoid-rich foods for their interventions, although the majority (*n = 5)* are cocoa based. One example of these studies, by Shin et al. (2022)^58^ supplemented participants with 30 g of either 70% or 85% cocoa chocolate for 3 weeks and found a significant reduction in negative affect scores following consumption of the 85% cocoa chocolate, which was not seen in the control (no chocolate) or 70% cocoa chocolate conditions. It is surprising that this difference was elicited between the 2 dark chocolate groups; however, the difference in polyphenols was large between the 2 groups, whereby the 70% group received 250 mg polyphenols and the 85% group received 500 mg cocoa polyphenols, which could contribute to changes in mood, as outlined in other cocoa studies with similar dosages. Furthermore, Shin et al. (2022)^58^ found significant differences in gut microbiota diversity between the control and 85% dark chocolate group, but not in the 70% dark chocolate group, suggesting a potential mechanistic link driving changes in mood between the 2 interventions.

#### Middle Aged Adults (36-65 years old)

Thirteen studies that fit the inclusion criteria had samples of middle-aged adults (35-65 years old), 6 (46%) of which found benefits of supplementing with whole flavonoids. Garrido et al. (2012)^30^ found after a 5-day Jerte Valley Cherry intervention that both middle aged and older adults had significantly reduced state anxiety compared to baseline. Four of the 6 of the studies found effects utilizing the STAI or Bond–Lader VAS, suggesting changes in anxiety and transient mood outcomes. Furthermore, 3 of the studies explored the effects of berries on mood, indicating that berry flavonoids may result in greater mood changes compared with other intervention food sources for this population. However, Lamport et al. (2016)^65^ investigated the effects of a 12-week concord grape juice intervention in this population and found no changes in mood using the STAI; however, as previously mentioned, this null effect may be due to the higher levels of stress and worse baseline mood state from the specific population of mothers with preteen children.

#### Older Adults (65+ years old)

Seven studies were identified in this review with a sample of older adults (65+ years old). Among these studies, only 1 study found benefits of whole flavonoid supplementation on mood and mental health for this population (Garrido et al. 2012^30^). All included studies had a chronic design, highlighting the need to explore acute mood effects of flavonoid-rich whole foods in older adults.

## DISCUSSION

This review aimed to evaluate the effects of flavonoids from the diet on mood and mental health, in healthy populations throughout the lifespan.

Overall, the evidence suggests that supplementation of study participants with flavonoid-rich foods in their whole or freeze-dried parts may benefit mood. However, further research with consistent methodology, such as carefully controlled dose–response studies and systematic approaches with clear rationales regarding choice of outcome measures is recommended. Consideration of bias within the studies is also important going forward, with more transparent recording of randomization procedures and handling of withdrawals as well as continuing to state clear sources of funding.

Duration of supplementation appears to be a key driver for benefits following this form of intervention, with chronic intake showing more consistent benefits compared to acute doses. However, when considering acute interventions the timing of intake and testing outcomes relative to peak metabolite levels are critical for observing changes in mood, which may be especially relevant for anthocyanin-rich interventions, for which effects may be dependent on absorption. As mentioned above, circulating anthocyanin metabolites have been found to peak between 1-2 and 6 hours, coinciding with changes in flow-mediated dilation,^80^ which could explain why anthocyanin-based interventions seem more likely to bring about acute benefits to mood. On the other hand, food sources such as cocoa may require chronic supplementation periods to result in changes in mood. These findings are indeed replicated when observing the cardiovascular effects of cocoa on mood, whereby improvements in vascular function are seen following chronic consumption of cocoa flavonoids.^81^^,^^82^ Cardiovascular and neuroprotective effects may emerge with sustained consumption of cocoa flavonoids, whereas the evidence for acute mood benefits following cocoa was less compelling. These findings highlight the need for further research to investigate the mechanisms of how flavonoids may benefit mood, while taking into account the different food sources and timeframes of supplementation. A better systematic approach to considering dosage effects is required. Currently, there is variability in the doses used across the trials, and it could be suggested that some standardization may be used in future research to better understand optimal intake levels for flavonoid-rich foods and mental health.

In this review we provide an updated overview of the effects of flavonoids on mood and mental health, with a focus on the benefits of flavonoid-rich foods consumed in their whole form, as opposed to flavonoid extracts. A limitation of this review is that it did not encompass all evidence on the effects of flavonoids on mood, as studies utilizing flavonoid extracts were excluded. While flavonoid extracts have been shown to benefit mood outcomes (Calapai et al. 2017)^71^), the strength of this evidence may be weaker (Jia et al. 2023).^21^ Additionally, the broader public health message of incorporating flavonoid-rich foods into the diet may not be fully represented by extract-based interventions.

A further limitation of this study is the absence of a meta-analysis. While a meta-analysis could have provided a more quantitative synthesis and strengthened the robustness of the findings, this was not feasible due to data-related constraints, including high heterogeneity across studies. Therefore, the review focused on a narrative synthesis of the included studies. Nevertheless, this review provides an updated perspective on the topic, which, in conjunction with prior meta-analyses in the field, contributes to the broader understanding of the effects of flavonoids on mood.

### Discussion of Findings for Trial Designs

The included studies in this review suggest that longer periods of supplementation of dietary flavonoids may benefit mood compared to a single acute dose. That is not to say, however, that flavonoids may not benefit mood acutely, although these acute effects seem to be greater when the intervention is anthocyanin based. Considering that the benefits can be found following a short period of supplementation, both considering chronic and acute trial designs, it could be suggested that flavonoids do not require habitual consumption to harbor benefits to mood. Instead, increasing flavonoid consumption at specific timepoints, when mood is expected to be poorer, may have greater benefits. For example, Baynham et al. (2021)^83^ suggested that a high-flavanol cocoa intervention (150 mg flavanols) attenuated a decline in endothelial function following a mental stress task, suggesting that a dose of flavonoids prior to mental stress may be physiologically beneficial. Furthermore, Herselman et al. (2022)^84^ reported a trend for improvement, whereby walnut consumption improved some aspects of mood during examination periods in university students. Subsequently, flavonoid-rich foods may be optimal for dosing during times of mental stress, although specific food item, dose, and length of dose for these specific events requires further research.

### Discussion of Findings by Food Source

Overall, the included studies reviewed suggest that increasing berries and cocoa in the diet may provide benefit to mood and mental health throughout the lifespan. However, the efficacy of the interventions on mood may depend on factors such as dosage of the intervention, whereby dosing between 30–774 mg berry anthocyanins, with an optimal dose around 250 mg; and approximately 500 mg cocoa flavonols may elicit optimal effects. This was also evident in the orange juice interventions, whereby 600 mg in the study by Park et al. (2020)^79^ led to significant changes in depression, which were not seen in the other orange juice interventions. As mentioned above, different sources of flavonoid-rich foods may also confer benefits over different timeframes, such as acute blueberry and anthocyanin-rich interventions and chronic cocoa interventions.

### Discussion of Findings by Age Group

This review highlights the finding that flavonoid supplementation may have effects on mood with varying efficacy across age groups and intervention types. Only 3 eligible studies involving children and adolescents were identified in the review, despite these suggesting benefits of flavonoids on mood in this cohort, the limited number of trials available make it difficult to draw firm conclusions, and these findings should be interpreted with caution. Conversely, young adults appear to benefit more from chronic interventions, particularly with high doses of anthocyanins. Middle-aged adults also show positive responses to flavonoid interventions, especially if the intervention is berry based. However, the older adult literature lacks acute intervention studies, suggesting that more acute designs are needed for this demographic. The evidence in the review also highlights the finding that specific population characteristics could also benefit more from flavonoid supplementation, such as individuals with low fruit and vegetable intake, which may be even more pronounced in young adults in whom diets are typically lower in fruit and vegetables,^85^ representing a larger opportunity for change.

### Measures

Mood measures were incredibly varied throughout the included studies, as shown in Table 3, although certain measures such as the STAI, which measures state and trait anxiety; Bond–Lader VAS, which assesses subjective mood states; and PANAS, which explores positive and negative affect, may suggest suitability for use in future dietary flavonoid and mood interventions due to the potential sensitivity to detect changes stemming from dietary interventions. In contrast, while the POMS, which measures dimensions of mood, and GDS, assessing depression in older adults, were commonly used instruments, they did not demonstrate significant effects in the context of flavonoid-rich food interventions.

**Table 3.** Number of studies reporting positive effect of a flavonoid rich food intervention versus control (effect/no effect) on mood outcomes.

Measure	Outcome	Effect/no effect	Citation
BDI	Depression	1/2	Krikorian et al. (2022); Sinclair et al. (2022), Velichkov et al. (2024) chronic data
BL-VAS	Transient affective state	3/5	Bondonno et al. (2014); Haskell-Ramsay et al. (2017); Lamport et al. (2020); Pase et al. (2013) (acute and chronic data); Coates et al. (2020); Kimble et al. (2022); Lamport et al. (2016)
CES-D	Depression	1/1	Park et al. (2020)
DASS	Depression & anxiety	1/1	Herselman et al. (2022)
GDS	Depression	0/3	Miller et al. (2018); Miller et al. (2021); Kok et al., (2005)
HRSD-17	Depression	1/1	Zhang et al. (2013)
MADRS	Depression	1/1	Zhang et al. (2013)
MFQ	Depression	1/1	Fisk et al. (2020)
MHC-SF	General mental health and wellbeing	1/1	Herselman et al. (2022)
PANAS	Transient affective state	7/7	Alharbi et al. (2016); Khalid et al.(2017) Study 1; Khalid et al.(2017) Study 2; Lamport et al. (2020); Whyte et al. (2019); Barfoot et al. (2021) a; Barfoot et al. (2021) b; Fisk et al. (2020); Kean et al. (2015); Shin et al. (2022); Simpson et al. (2019); Tsang et al. (2019); Velichkov et al. (2024) acute data; Colombage et al. (2024)
PHQ	Depression	0/2	Barfoot et al. (2021) b; Velichkov et al. (2024) chronic data
POMS	Transient affective state	0/7	Boolani et al. (2017); Marsh et al. (2017); Coates et al. (2020); Pribis. (2016); Herselman et al. (2022); Miller et al. (2018); Miller et al. (2021)
RCADS	Depression & anxiety	0/1	Fisk et al. (2020)
SF-36	General mental health and wellbeing	0/1	Kok et al. (2005)
STAI	Anxiety	4/4	Martin et al. (2012); Scholey et al. (2010); Abdelhalim (2021); Barfoot et al. (2021) b; Garrido et al. (2012); Lamport et al. (2016); Sinclair et al. (2022); Colombage et al. (2024)
GAD-7	Anxiety	0/1	Velichkov et al. (2024) chronic data
SHAPS	Depression	0/1	Velichkov et al. (2024) chronic data
EPDS	Depression (postpartum)	0/1	Colombage et al. (2024)
PSAS-RSF-C	Anxiety (postpartum)	0/1	Colombage et al. (2024)
WHOQOL-BREF	General mental health and wellbeing	1/1	Barfoot et al. (2021) b; Colombage et al. (2024)

Abbreviations: BDI, Beck Depression Inventory; BL-VAS, Bond–Lader Visual Analogue Scale; CES-D, Center for Epidemiologic Studies Depression Scale; DASS, Depression, Anxiety, and Stress Scale; EPDS, Edinburgh Postnatal Depression Scale; GAD-7, Generalized Anxiety Disorder 7-item scale; GDS, Geriatric Depression Scale; HRSD-17, Hamilton Rating Scale for Depression, 17-item; MADRS, Montgomery–Åsberg Depression Rating Scale; MFQ, Mood and Feelings Questionnaire; MHC-SF, Mental Health Continuum–Short Form; PANAS, Positive and Negative Affect Schedule; PHQ, Patient Health Questionnaire; POMS, Profile of Mood States; PSAS-RSF-C, Postnatal Specific Anxiety Scale Research Short Form for global Crisis; RCADS, Revised Child Anxiety and Depression Scale; SF-36, Short Form Health Survey, 36 items; STAI, State-Trait Anxiety Inventory; SHAPS, Snaith-Hamilton Pleasure Scale; WHOQOL-BREF, World Health Organisation Quality Of Life Brief Version.

## CONCLUSIONS

The impact of flavonoids within the diet on mood and mental health looks promising, although it may vary by age group and dietary habits. Younger populations, particularly adolescents and young adults, benefit more noticeably from chronic interventions, whereas older adults show less pronounced effects. These findings may be due to the way younger and older adults perceive their mood, with evidence suggesting that older adults feel happier on average and have different emotion regulation strategies compared to younger adults,^86^^,^^87^ indicating a larger scope for change in mood ratings as a result of dietary interventions. Additionally, individuals with lower baseline fruit and vegetable intake might experience more significant mood enhancements from flavonoid supplementation, highlighting a potential area for dietary improvement. Finally, measures like the STAI, Bond–Lader VAS, and PANAS may offer good sensitivity to changes induced by dietary interventions, suggesting their suitability for future studies on this topic.

Overall, while flavonoid supplementation shows promise for mood and mental health, particularly with chronic intake and specific sources of flavonoid foods, further research is needed to refine dosing strategies and mechanisms of action and explore the effects across different populations and intervention durations.

## Supplementary Material

nuaf188_Supplementary_Data

## Data Availability

The data utilised for all analyses were obtained from the included studies and are publicly accessible.

## Supplementary Material

Supplementary Material 1, PRISMA checklist; Supplementary Material 2, quality criteria checklist results for each study; Supplementary Material 3, Search terms.
