# Supplementary material for: Effects of Dietary Flavonoids on Mood and Mental Health: A Systematic Review
Source: Nutr Rev. 2025 Nov 14;84(4):734–50. doi: 10.1093/nutrit/nuaf188 (PMC13017404; doi:10.1093/nutrit/nuaf188)

PRISMA 2020 flow diagram for new systematic reviews which included searches of databases, registers and other sources

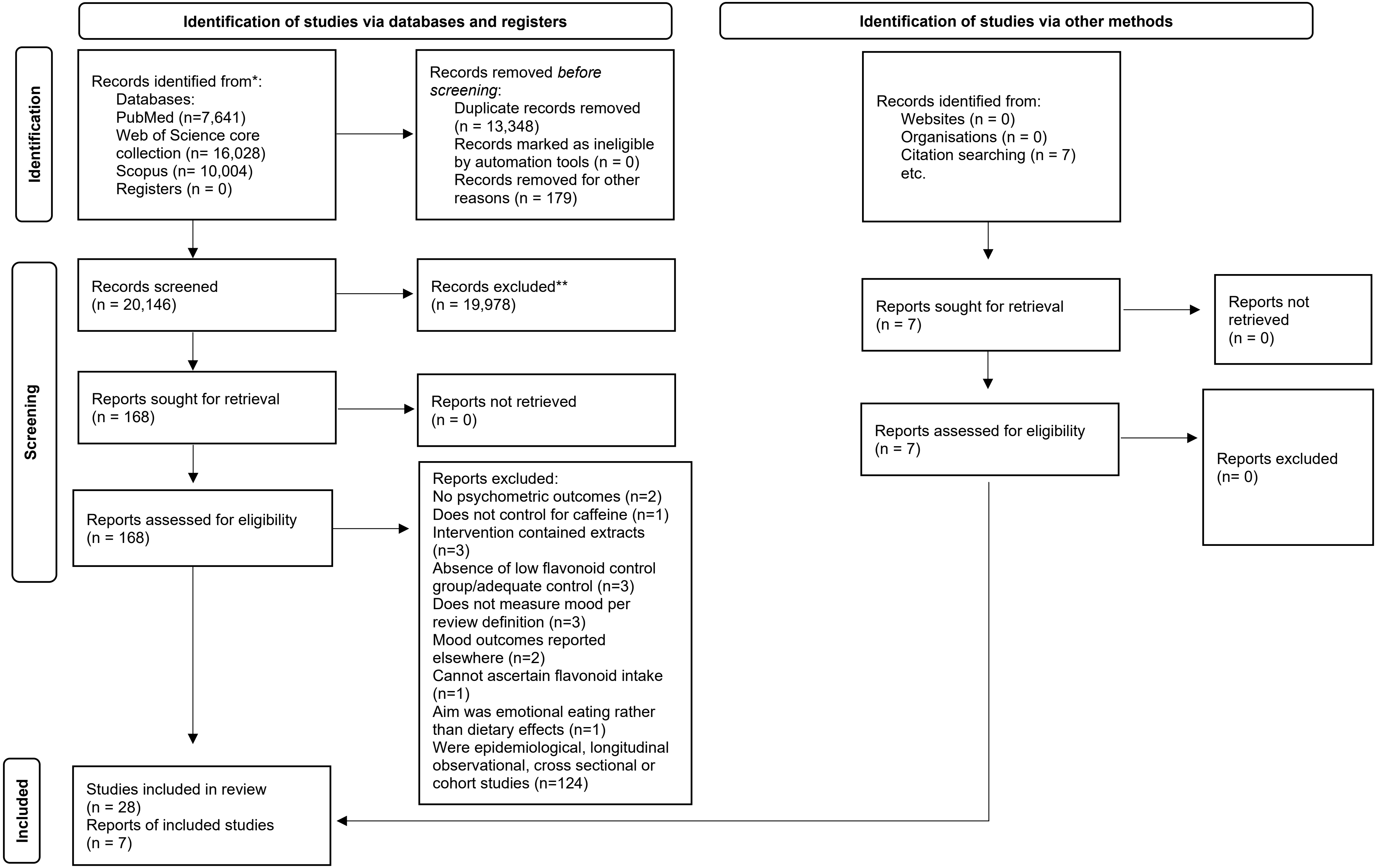

Supplement: nuaf188_Supplementary_Data [file nuaf188_supplementary_data.zip]
